# Development and Validation of a Nomogram to Predict Survival in Pancreatic Head Ductal Adenocarcinoma After Pancreaticoduodenectomy

**DOI:** 10.3389/fonc.2021.734673

**Published:** 2021-09-29

**Authors:** Feng Peng, Tingting Qin, Min Wang, Hebin Wang, Chao Dang, Chien-Hui Wu, Yu-Wen Tien, Renyi Qin

**Affiliations:** ^1^ Department of Biliary-Pancreatic Surgery, Affiliated Tongji Hospital, Tongji Medical College, Huazhong University of Science and Technology, Wuhan, China; ^2^ Department of Surgery, National Taiwan University Hospital, Taipei, Taiwan

**Keywords:** pancreatic head duct adenocarcinoma, nomogram, prognosis, curative resection, overall survival

## Abstract

**Background:**

Pancreatic head ductal adenocarcinoma (PHDAC) patients with the same tumor-node-metastasis (TNM) stage may share different outcomes after pancreaticoduodenectomy (PD). Therefore, a novel method to identify patients with poor prognosis after PD is urgently needed. We aimed to develop a nomogram to estimate survival in PHDAC after PD.

**Methods:**

To estimate survival after PD, a nomogram was developed using the Tongji Pancreatic cancer cohort comprising 355 PHDAC patients who underwent PD. The nomogram was validated under the same conditions in another cohort (*N* = 161) from the National Taiwan University Hospital. Prognostic factors were assessed using LASSO and multivariate Cox regression models. The nomogram was internally validated using bootstrap resampling and then externally validated. Performance was assessed using concordance index (c-index) and calibration curve. Clinical utility was evaluated using decision curve analysis (DCA), X-tile program, and Kaplan–Meier curve in both training and validation cohorts.

**Results:**

Overall, the median follow-up duration was 32.17 months, with 199 deaths (64.82%) in the training cohort. Variables included in the nomogram were age, preoperative CA 19-9 levels, adjuvant chemotherapy, Tongji classification, T stage, N stage, and differentiation degree. Harrell’s c-indices in the internal and external validation cohorts were 0.79 (95% confidence interval [CI], 0.76–0.82) and 0.83 (95% CI, 0.78–0.87), respectively, which were higher than those in other staging systems. DCA showed better clinical utility.

**Conclusion:**

The nomogram was better than TNM stage and Tongji classification in predicting PHDAC patients’ prognosis and may improve prognosis-based selection of patients who would benefit from PD.

## Introduction

Pancreatic head ductal adenocarcinoma (PHDAC) is one of the most lethal cancers of the digestive system (1, 2). Despite improvements in diagnosis, surgical techniques, and comprehensive treatment with follow-up, PHDAC remains an intractable disease with a global 5-year survival rate of 3%–15% (3, 4). Pancreaticoduodenectomy (PD) is the primary option to improve long-term survival in PHDAC patients (5). However, due to insufficient individualized intervention, the outcomes were diverse after surgical intervention (6, 7). Thus, it is essential to predict precise prognosis of PHDAC patients after PD to guide early individualized treatment.

Tumor-node-metastasis (TNM) staging is a widely used predictive system to guide surgical intervention and indicate postoperative prognosis in PHDAC (8), hence its derived American Joint Commission on Cancer (AJCC) clinical staging system and the Japanese Pancreas Society (JPS) classification. However, these staging systems just rely on anatomical and pathological features regardless of patients’ clinical characteristics or the tumor’s biological characteristics (9) and have inherent limitations to predict the prognosis of PHDAC (8, 10–12). Furthermore, in the eighth AJCC stage, PHDAC with the celiac axis, superior mesenteric artery (SMA), and/or common hepatic artery involved was defined as being in the T4 stage, which failed to demonstrate the situation of superior mesenteric vein/portal vein (SMV/PV) invasion. The idiographic situation of vessel invasion decides the choice of upfront treatment. The Tongji classification (TJC) is an efficient classification system to indicate the vasculature and the choice of surgical approach (13). It is a good supplement of AJCC stage to guide upfront treatment of PHDAC.

Over the years, integrated predictive systems, such as nomogram (14) combined with multiple predictors, proved to have higher predictive accuracies than that of a single predictor (15). Many useful nomograms had been developed to predict the prognosis of pancreatic ductal adenocarcinoma (PDAC), which included all types of PDAC together and without considering the tumor’s location (16, 17). Tumors located in different parts of the pancreas (head, body, and tail) might have different biological properties; require different diagnoses, surgical options, and perioperative treatments; and show different outcomes after surgery. The morbidity of PHDAC was higher than those of pancreatic body and tails (18). The PDAC locations in body and tail are larger, more often metastasized, and less often resectable than in the pancreatic head (19). However, the comparison of two cohorts of prognosis was controversial (20, 21). Therefore, it is necessary to establish an integrated predictive model for evaluating the prognosis in PHDAC after PD to increase the basis of treatment selection.

In this study, we aimed to establish and externally validate an effective nomogram that integrated clinicopathological characteristics and perioperative features to accurately predict the individual survival after surgery in PHDAC patients. To the best of our knowledge, this is the first report of a nomogram that was developed and validated to predict the survival of PHDAC patients after PD.

## Methods

### Patients and Database Description

This retrospective study included 355 PHDAC patients who underwent PD between January 2012 and June 2018 at Tongji Hospital, Wuhan, China. Data were extracted from a computerized database, which was a registry comprising data of more than 1,600 patients who underwent pancreatic surgery since January 1, 2012. It consecutively documents patients’ medical records and the follow-up data in the Institute of Biliary-Pancreatic Surgery, Tongji Hospital. To examine the generalizability of the model, an external validation cohort comprising 161 PHDAC patients who underwent PD during 2005–2020 was set at the National Taiwan University Hospital. All patients admitted to our surgical department would first receive an assessment of surgical resectability by the multi-disciplinary team (MDT) through imaging to ascertain as curatively resectable PDAC preoperatively, even with peripancreatic invasion or artery (hepatic, superior mesenteric, and celiac artery) or vein (portal or superior and inferior mesenteric vein) that could be completely resected and reconstructed. Those who were evaluated as unresectable will be transferred to the oncology department for neochemotherapy. Therefore, patients were included if (i) they had their first visit and underwent PD; (ii) their tumors are located in the head of the pancreas; and (iii) their tumors are histopathologically confirmed as PDAC. Those who had missing values in the follow-up data or with a missing rate more than 20% were excluded. Moreover, to avoid inclusion of deaths due to postoperative complications, patients who died within 30 days of surgery were excluded when analyzing long-term outcomes. All patients received standard postoperative care and reached functional recovery (criteria: independent mobility, ability to maintain at least 50% of the daily required caloric intake, no signs of infection, and no need for intravenous fluid). This study was approved by the Institutional Review Board of Tongji Medical College, Huazhong Scientific and Technological University, China, and the Ethics Committee of the National Taiwan University Hospital. The requirement of written informed consent was waived by the Institutional Review Board.

### Study Variables and Definition

A standardized data form was created to retrieve all relevant information on clinicopathological (including demographic, preoperative, intraoperative, and postoperative variables) and oncological features. Demographic information included sex, age, body mass index (BMI), smoking/drinking habits, and history of diabetes. The 7th edition JPS classification and AJCC 8th edition clinical staging system for pancreatic adenocarcinoma were evaluated (22, 23). The TJC, proposed by Prof. Qin, is described in detail elsewhere (13); it aims to help surgeons in undertaking an individualized surgical approach for patients with different types of vascular invasion. The four types of invasion in TJC are as follows: Type I: PHDAC without vascular invasion; Type II: PHDAC with SMV/PV invasion but no SMA invasion; Type III: PHDAC without SMV/PV invasion or compression but with <180° suppression or invasion of the SMA; and Type IV: PHDAC with SMV/PV and SMA involvement or compression <180°. Operation duration was defined as the time from skin incision or trocar placement to complete skin closure. Estimated intraoperative blood loss (EIBL) was carefully recorded by the anesthetist using a vacuum system. Postoperative length of stay (LOS) was defined as the number of days from operation to discharge. Postoperative comorbidities, including postpancreatectomy hemorrhage, pancreatic leakage, and delayed gastric emptying, were defined according to the International Study Group of Pancreatic Surgery definition (24). Tumor size was defined as the maximal diameter of the tumor in the resected specimen. Resection margin status (negative [R0] or microscopically positive [R1]) was ascertained based on the final pathological assessment. The primary observation index was OS after PD, which was calculated as the duration between the date of surgery to death by any cause (or the last documented follow-up with no death).

### Statistical Analysis

Data of continuous variables are presented as mean and standard deviation or median and interquartile range (IQR) for skewed distribution. Data of categorical variables are expressed as frequencies and percentages. Continuous variables were transformed into categorical variables based on recognized cutoff values (for BMI, preoperative blood test indicators, tumor markers, including CA 19-9, CEA, and tumor size), or the tertiles of respective levels (for operation duration, intraoperative blood loss, and number of lymph nodes obtained). Variables with <20% missing values were imputed using a non-parametric imputation method, MissForest.

Optimal features were selected using the least absolute shrinkage and selection operator (LASSO) Cox regression model, and factors with nonzero coefficients were identified and selected (25). The selected features were incorporated into the Cox regression analysis, following Harrell’s guidelines, to evaluate associations of relevant variables with OS. Hazard ratios (HRs) and 95% confidence interval (CI) were calculated. Based on the results of the Cox model, a nomogram was developed to predict the probability of 1-, 2-, and 3-year OS rates after surgical resection. For allocating points in the nomogram, regression coefficients were applied to each observation to define the linear predictor of survival probability. The model’s performance was evaluated based on the discriminating ability (discrimination) and accuracy of point estimates of the survival function (calibration) with 1,000 time bootstraps. Moreover, clinical utilities of the nomogram were carefully investigated using the decision curve analysis (DCA). By grouping patients evenly into different risk groups within a certain AJCC stage, according to the total risk scores (from the highest to lowest) in the training cohort, we used the X-tile program with the minimum *p*-value to determine optimal cutoff values. These values were further applied to the validation cohort, and the respective Kaplan–Meier survival curves were constructed. All data management and statistical analyses were performed using SAS 9.4 (SAS Institute, Inc., USA) and R software packages (version 3.6.0, http://www.r-project.org). All statistical tests were two-sided, and *p* < 0.05 was considered statistically significant.

## Results

### Patient Characteristics in the Training and Validation Cohorts

In the training cohort, the data of 355 PHDAC patients who underwent PD or total pancreatectomy during the study period were extracted; of them, 307 patients met the inclusion/exclusion criteria and were enrolled in the study. The median age of patients was 58 (range, 24–82) years, and 60.26% (185/307) of patients were men. Surgical treatment was performed between January 2012 and June 2018, and the last follow-up was performed before June 2021. There were 199 deaths (64.82%) over a median follow-up duration of 32.17 months (IQR, 24.93–44.47 months). The median survival duration was 23.73 months (95% CI, 22.1–26.77 months). The validation cohort comprised the entire 161 patients; of them, 159 were diagnosed with PHDAC between July 2005 and January 2021 at the National Taiwan University Hospital. Their median age was 66 (range, 28–95) years; 57.23% (91/159) of patients were men, and 90.2% (142/159) underwent PD. There were 67 deaths during a median follow-up period of 18.0 months (IQR, 10~27 months), and the median survival was 21.1 months (95% CI, 15.9–28.8 months). Furthermore, the median LOS was 21 (IQR, 16–27) days in the Tongji cohort and 23.00 (IQR, 16.00–33.00) days in the Taiwan cohort. In total, 22.15% (68/307) patients presented with postoperative complications in the Tongji cohort and 25.79% (41/159) in the Taiwan cohort. Patients in the training set with TJC Type I and II were better than those with Type III/IV (mOS: 28.37 months, 95% CI, 24.17–31.37 in Type I, versus 18.4 months, 95% CI, 16.8–23.07 months in Type II, and versus 11.83 months, 95% CI, 9.4–16.4 months in Type III/IV, *p* < 0.0001, **Supplementary Figure 1A**). In the validation set, patients with TJC Type I and II were better than those with Type III/IV (mOS: 25 months, 95% CI, 18.4–NA in Type I, versus 16.6 months, 95% CI, 11.0–29.2 months in Type II, and versus 7.7 months, 95% CI, 5.9–NA months in Type III/IV, *p* < 0.0001, **Supplementary Figure 1B**). The clinicopathological and pre- and postoperative characteristics of patients in the training and validation cohorts are listed in **Table 1**.

**Table 1:** Demographic, clinicopathological, and pre- and postoperative characteristics of pancreatic head cancer patients in the training and validation cohorts.

Characteristics	Training cohort (*N* = 307)	Validation cohort (*N* = 159)
Age, years, median (range)	58.00 (24.00–82.00)	66.00 (28.00–85.00)
Sex, *N* (%)		
Male	185 (60.26)	91 (57.23)
Female	122 (39.74)	68 (42.77)
BMI level, kg/m^2^, median (range)	21.45 (14.84–36.23)	22.60 (15.43–31.04)
18.5–23 kg/m^2^, *N* (%)	180 (58.63)	76 (47.8)
<18.5 kg/m^2^, *N* (%)	37 (12.05)	14 (8.81)
≥23 kg/m^2^, *N* (%)	90 (29.32)	69 (43.4)
Smoking habit, *N* (%)	64 (20.85)	74 (46.54)
History of diabetes mellitus, *N* (%)	98 (31.92)	72 (45.28)
Tumor size, cm, median (range)	3.00 (1.00–9.60)	3.20 (1.50–13.00)
≤2, *N* (%)	59 (19.22)	10 (6.29)
3–4, *N* (%)	211 (68.73)	116 (72.96)
≥4, *N* (%)	37 (12.05)	33 (20.75)
Tumor differentiation, *N* (%)		
Well	41 (13.36)	18 (11.32)
Moderate	146 (47.56)	123 (77.36)
Poor	120 (39.09)	18 (11.32)
CA 19-9, U/ml, median (range)	157.90 (0.60–12,000.00)	395.50 (1.00–26,000.00)
<160, *N* (%)	154 (50.16)	59 (37.11)
160–400, *N* (%)	52 (16.94)	21 (13.21)
≥400, *N* (%)	101 (32.9)	79 (49.69)
CEA, ng/ml, median (range)	3.10 (0.50–1,255.99)	2.75 (0.10–140.60)
≤5.9, *N* (%)	249 (80.06)	118 (74.21)
>5.9, *N* (%)	62 (19.94)	41 (25.79)
Operation time, min, median (range)	390.00 (180.00–720.00)	267.00 (179.00–599.00)
≤300, *N* (%)	91 (29.64)	116 (72.96)
300–500, *N* (%)	180 (58.63)	42 (26.42)
>500, *N* (%)	36 (11.73)	1 (0.63)
EIBL, ml, median (range)	400.00 (10.00–3,500.00)	422.25 (10.00–2,120.00)
<500, *N* (%)	164 (53.42)	88 (55.35)
500–1000, *N* (%)	81 (26.38)	52 (32.70)
>1000, *N* (%)	62 (20.20)	19 (11.95)
Lymph node, median (range)	17.00 (1.00–67.00)	18.00 (1.00–60.00)
<3, *N* (%)	16 (5.21)	7 (4.40)
3–12, *N* (%)	57 (18.56)	27 (16.98)
>12, *N* (%)	234 (76.22)	125 (78.62)
Positive lymph nodes, median (range)	0.00 (0.00–11.00)	2.00 (0.00–14.00)
0, *N* (%)	201 (65.47)	43 (27.04)
0–3, *N* (%)	82 (26.71)	88 (55.35)
>3, *N* (%)	24 (7.82)	28 (17.61)
Surgical margin		
R0, *N* (%)	263 (85.67)	103 (64.78)
R1, *N* (%)	44 (14.33)	56 (35.22)
JPS, *N* (%)		
IA	23 (7.4)	4 (2.52)
IB	167 (53.7)	31 (19.5)
IIA	12 (3.86)	9 (5.66)
IIB	109 (35.05)	115 (72.33)
T stage, *N* (%)		
1	54 (17.59)	10 (6.29)
2	216 (70.36)	115 (72.33)
3	37 (12.05)	34 (21.38)
N stage, *N* (%)		
0	201 (65.47)	43 (27.04)
1	82 (26.71)	88 (55.35)
2	24 (7.82)	28 (17.61)
AJCC stage, *N* (%)		
IA	42 (13.68)	5 (3.14)
IB	138 (44.95)	35 (22.01)
IIA	21 (6.84)	3 (1.89)
IIB	82 (26.71)	88 (55.35)
III	24 (7.82)	28 (17.61)
Combined vasectomy, *N* (%)	67 (21.82)	74 (46.54)
Tongji classification, *N* (%)		
Type I	158 (51.47)	92 (57.86)
Type II	125 (40.72)	55 (34.59)
Type III+IV	24 (7.81)	12 (7.55)
Chemotherapy, *N* (%)	209 (68.08)	118 (74.21)
CD ≥ 3, *N* (%)	68 (22.15)	41 (25.79)
LOS, days, median (range)	22.00 (7.00–64.00)	23.00 (8.00–86.00)

BMI, body mass index; WBC, white blood cell; RBC, red blood cell; CEA, carcinoma embryonic antigen; EIBL, estimated intraoperative blood loss; CA 19-9, cancer antigen 19-9; JPS, Japanese Pancreas Society; AJCC, American Joint Committee on Cancer; LOS, length of stay; CD, Clavien-Dindo.

### Prognostic Feature Selection With LASSO Analysis in the Training Cohort

**Figure 1:**
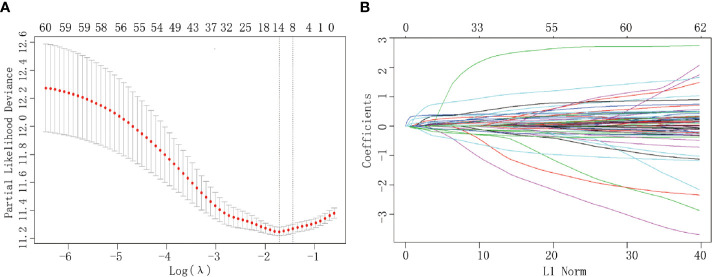
Clinicopathological parameter identification and feature selection using the LASSO regression model. **(A)** Tenfold cross-validation was applied to select the most suitable feature using the LASSO COX regression model. **(B)** Coefficient curves for the 62 parameters.

Of all the demographic, laboratory examination, and clinicopathological variables, 14 features were selected out of 62 based on the LASSO Cox regression model (**Figures 1A, B**). The analysis indicated that age, BMI level, tumor size, preoperative platelet and CA 19-9 levels, N stage, T stage, AJCC stage, TJC, surgical margin, intraoperative blood loss, operation duration, adjuvant chemotherapy, and tumor differentiation were associated with prognosis. All significant factors selected from the LASSO Cox model were further analyzed using the multivariable Cox regression model. Age, elevated preoperative CA 19-9 levels, N stage, T stage, TJC, tumor differentiation degree, and adjuvant chemotherapy were independent prognostic factors in the multivariable Cox model (**Table 2**).

**Table 2:** Univariate analysis (LASSO Cox) and multivariate Cox proportional hazards regression analysis of the primary cohort.

Variable	Univariate LASSO Cox analysis		Multivariate analysis	
	Hazard ratio (95% CI)	*p*-value	Hazard ratio (95% CI)	*p*-value
Age	1.00 (0.99–1.02)	0.085	1.02 (1.01–1.03)	0.046
BMI level, kg/m^2^				
18.5–23	Reference			
<18.5	1.05 (0.68–1.60)	0.836		
≥23	0.71 (0.51–0.98)	0.038		
Tumor size, cm	1.14 (1.02–1.28	0.024		
Reduced preoperative platelets (≤100 × 10^9^/L)	1.90 (0.89–4.05)	0.097		
Preoperative CA 19-9, U/ml				
<160	Reference		Reference	
160–400	1.39 (0.94–2.04)	0.097	1.48 (0.98–2.22)	0.060
≥400	1.62 (1.18–2.23)	0.003	1.54 (1.12–2.15)	0.010
T Stage				
T1	Reference		Reference	
T2	1.31 (0.881.94)	0.186	1.26 (0.83–1.90)	0.273
T3	1.89 (1.13–3.16)	0.015	2.20 (1.26–3.84)	0.005
N Stage				
N0	Reference		Reference	
N1	1.48 (1.08–2.04)	0.016	1.29 (0.92–1.82)	0.142
N2	2.94 (1.72–5.01)	<0.001	3.66 (2.10–6.35)	<0.001
AJCC stage				
IA	Reference			
IB	1.12 (0.71–1.77)	0.615		
IIA	2.14 (1.13–4.06)	0.019		
IIB	1.74 (1.07–2.81)	0.026		
III	3.49 (1.83–6.65)	<0.001		
Surgical margin				
R0	Reference			
R1	1.80 (1.22–2.66)	0.003		
EIBL, ml				
<200	Reference			
200–500	1.17 (0.760–1.79)	0.484		
≥500	1.51 (1.008–2.26)	0.046		
Tongji classification				
Type I	Reference		Reference	
Type II	1.26 (0.938–1.70)	0.125	1.13 (0.82–1.56)	0.444
Type III	3.51 (1.531–8.04)	0.003	3.62 (1.50–8.74)	0.004
Type IV	2.83 (1.604–4.98)	<0.001	2.91 (1.59–5.31)	<0.001
Operation time, min				
≤300	Reference			
300–500	1.34 (0.939–1.90)	0.107		
>500	2.05 (1.268–3.32)	0.003		
Tumor differentiation				
Well	Reference		Reference	
Moderate	2.25 (1.28–3.96)	0.005	1.87 (1.04–3.37)	0.038
Poor	2.87 (1.63–5.04)	<0.001	2.34 (1.30–4.22)	0.005
Chemotherapy	0.20 (0.14–0.27)	<0.001	0.19 (0.13–0.27)	<0.001

EIBL, estimated intraoperative blood loss; AJCC, American Joint Committee on Cancer; CA 19-9, cancer antigen 19-9; BMI, body mass index; CI, confidence interval.

T stage and N stage were inputted into the multivariate model instead of the AJCC stage to avoid the multicollinearity.

### Prognostic Nomogram for OS

**Figure 2:**
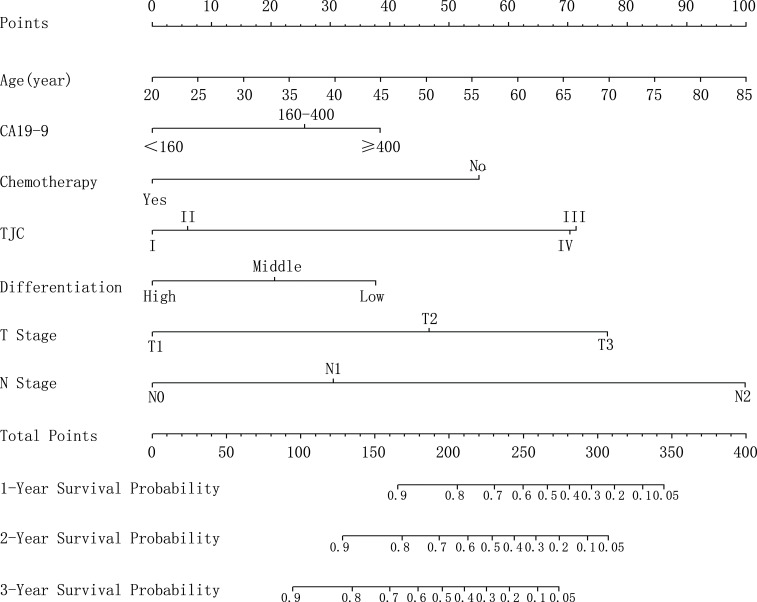
The developed nomogram for predicting overall survival. The nomogram was developed based on the training cohort, with the use of age, preoperative CA19-9 levels, adjuvant chemotherapy, Tongji classification, differentiation degree, T stage, and N stage. CA 19-9, cancer antigen 19-9.

As shown in **Figure 2**, the nomogram that included factors based on the multivariate Cox regression analysis was established. The nomogram illustrated that age and N stage shared the largest contributions to patient prognosis, followed by T stage and TJC. Concurrent adjuvant chemotherapy, preoperative CA 19-9 levels, and tumor differentiation degree showed a moderate effect on patient’s survival. The specific points of each predictor are shown in **Supplementary Table 1**. By summing up the total score and locating it on the total point scale, a straight line can be drawn to estimate the probability of survival at each time point.

### Calibration and Validation of Predictive Accuracy of the Nomogram for OS

**Figure 3:**
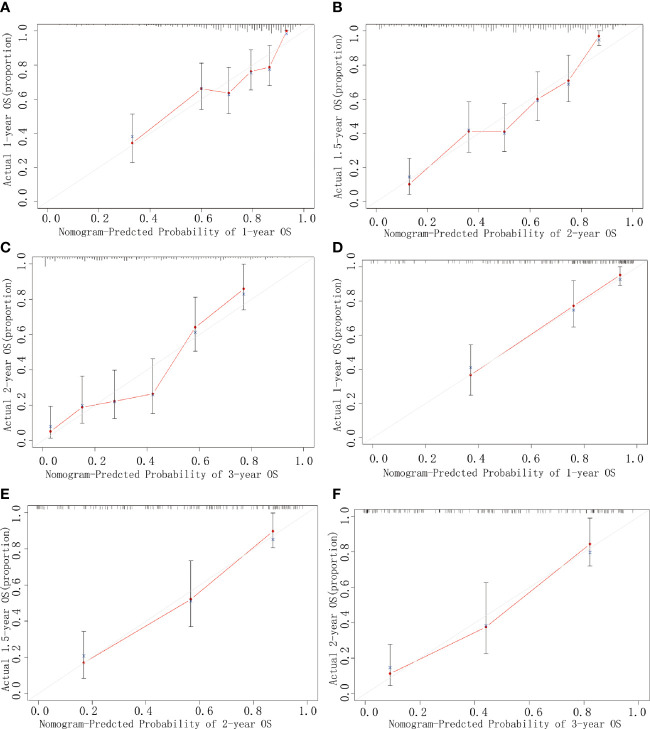
Calibration curves. Curves for 1-year **(A)**, 2-year **(B)**, and 3-year **(C)** OS in the training cohort, and those for 1-year **(D)**, 2-year **(E)**, and 3-year **(F)** OS in the validation cohort. The predicted possibility of the year-specific OS rate is indicated in the *x*-axis, and the actual possibility of the year-specific OS rate is indicated in the *y*-axis.

The calibration plots using 1000 sets of simulated data through bootstrapping presented an acceptable agreement in the training cohort and excellent agreement in the validation cohort between nomogram prediction and actual observation for 1-, 2-, and 3-year OS (**Figures 3A–F**). Moreover, the Harrell’s c-index for the established nomogram to predict OS was 0.79 (95% CI, 0.76–0.82) in the training cohort and 0.83 (95% CI, 0.78–0.87) in the validation cohort. Altogether, these results suggest good discriminative and predictive abilities of the nomogram in predicting survival in PHDAC patients. Additionally, as summarized in **Supplementary Table 2**, the established nomogram displayed a better Harrell’s c-index in predicting survival than any single independent prognostic factor in both training cohort and validation cohort.

### Clinical Utility of the Nomogram

**Figure 4:**
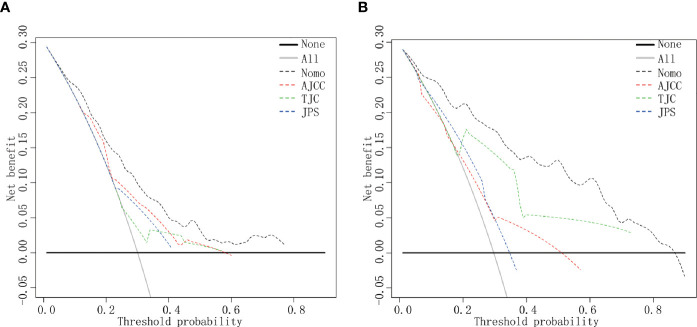
DCA curves. DCA curves in the training cohort **(A)** and the validation cohort **(B)**. Clinical usefulness of different predictive systems in predicting overall survival at various time points. The *y*-axis represents net benefit. The *x*-axis shows threshold probability. The black dotted line displays the benefit of the developed nomogram. The red dotted line displays the benefit of the AJCC stage. The green dotted line displays the benefit of Tongji classification, and the blue dotted line displays the JPS stage. Nomo, the developed nomogram model.

**Figure 5:**
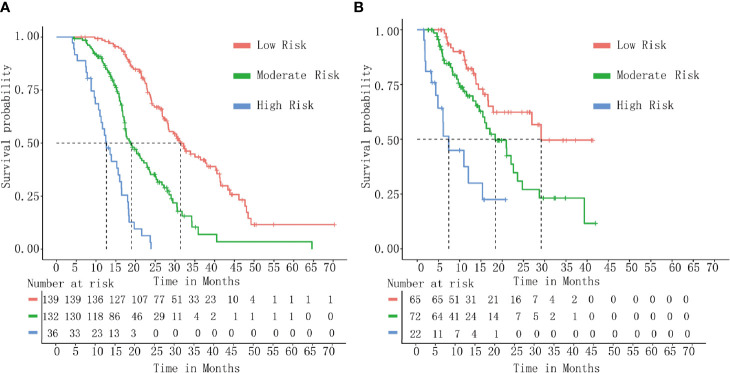
Kaplan–Meier curve analysis. Survival curves stratified by the risk score calculated using the developed prediction model [(low risk (<176), moderate risk (176–256), and high risk (>256)] in the training cohort **(A)** and the validation cohort **(B)**.

DCA indicated that the nomogram provided superior net benefit than the commonly used international staging system and TJC in both the training (Tongji) and validation (Taiwan) cohorts (**Figures 4A, B**). We further determined the cutoff values to predict mortality by grouping patients in the training cohort into three risk subgroups, namely, low risk (<176), moderate risk (176–256), and high risk (>256), according to the cutoff points determined by the X-tile analysis (**Supplementary Figure 2**). The Kaplan–Meier curves constructed for both the training and validation cohorts indicated that the nomogram could predict the probability of survival in PHDAC patients post PD (**Figure 5**). Moreover, after applying cutoff values to patient subgroups at different AJCC stages in the training cohort, the nomogram risk model showed good discriminative ability in predicting survival outcomes within stage I (log-rank *p* < 0.01), stage II (log-rank *p* < 0.01), and stage III (log-rank *p* < 0.01) (**Supplementary Figures 3A–C**). In the validation cohort, applying the cutoff values in different AJCC stages to patient subgroups also allowed significant distinction between Kaplan–Meier curves for survival outcomes in stages I, II, and III (**Supplementary Figures 3D–F**).

## Discussion

Prediction survival is crucial in oncology. In this study, we established and validated a nomogram model for PHDAC patients to accurately predict the long-term prognosis by combining simple clinicopathological factors, which showed good performance in both the training and validation cohorts. As PHDAC patients show poor long-term survival, an accurate prediction of prognosis in patients after surgery is of increased clinical significance. We anticipate that this practical predictive tool can potentially guide individualized therapy, as surgeons can predict the prognosis of PHDAC patients after PD precisely and the early intervention can be given presciently.

Predictive factors used in our nomogram can be readily ascertained from clinical information, making it feasible for application in clinical practice. Upfront treatment choice is the most important prognosis factor in any kind of cancer. In our nomogram, the TJC type was an effective surgical guideline to cope with different anatomical relationships observed with vascular invasion in PHDAC. Different vascular invasion (SMA/SMV/PV) may result in different tumor metastasis pathways and different prognosis, which could be illustrated clearly by different TJC types. Not all those borderline resectable PHDAC (TJC type II/III/IV) could convert to surgery after neoadjuvant therapy. Based on the existing evidence, patients with borderline resectable PHDAC should be treated depending on the type of vascular involvement. In case of venous borderline PHDAC complete tumor removal combined with venous replacement is the treatment of choice, while in arterial borderline PHDAC, the decision for upfront resection must be made more critically due to reduced rates of R0 resections, among other aspects (26, 27). What is more, considering some patients may refuse to receive neoadjuvant chemotherapy due to various reasons, PD combined with blood vessel reconstruction and postoperative adjuvant chemotherapy may be their better choice. Moreover, concerns for tumor progression or holistic functional deterioration during the course of neoadjuvant therapy and low conversion rates from neoadjuvant therapy to surgery can lead to the loss of a ‘‘window of opportunity’’ during which the patient may have had a radical surgery. For example, in the SWOG S1505 trial, 25% of the 102 eligible patients who received 3 months of preoperative mFOLFIRINOX or gemcitabine plus albumin-bound paclitaxel failed to proceed to surgery due to disease progression and chemotherapy-related toxic effects. Four patients proceeded to surgery while did not undergo a resection because of metastatic disease or complications (28). Thus, the upfront PD might be an appropriate treatment option for those PHDAC cases (with different TJC type) that could not benefit from neoadjuvant therapy. Therefore, we focus on patients who received upfront PD to investigate the correlation between surgery and survival benefit.

TJC is used primordially as a guidance for selecting a suitable surgical approach in PD (13). The clinical outcomes of different surgical approaches (including SMA as the first approach or vein involvement approach) in PD were inconsistent. For example, some studies showed that the SMA-first approach had better clinical outcomes, particularly regarding longer survival in PDAC patients (29), while other studies demonstrated no difference in long-term survival in patients undergoing different PD approaches (30). The survival curves of PHDAC patients showed distinct differences among different TJC types. It remains an interesting topic that needs to be further investigated in our next study with more robust data.

Our nomogram achieved a c-index of 0.79 in the training cohort and 0.83 in the external validation cohort, indicating a good performance in distinguishing patients with different outcomes. Additionally, the calibration curve demonstrated good accuracy of the model in predicting survival in PHDAC patients, which was significantly superior to the AJCC stage. Currently, the TNM staging system is the widely used standard for predicting OS in oncology, while its application in predicting patients’ survival has unavoidable drawbacks (8). For example, patients with the same TNM stage may have different prognoses. In our study, the predictive accuracy (measured by Harrell’s c-index) of different staging systems was varied from 0.57 to 0.66 in the training cohort and from 0.53 to 0.68 in the validation cohort, significantly lower than that of nomogram in both the cohorts, respectively. Additionally, the nomogram showed good discriminative ability by stratifying patients into three risk groups overall, or in separate AJCC stages I, II, and III, respectively. Thus, we can further identify high-risk patients with worse stages after surgery through this nomogram tool.

An increased usage of nomograms for predicting prognosis in PDAC patients indicates their importance in this field. The recent nomogram developed by Oba et al. (17), based on preoperative objective variables for PDAC, could be used to assess the probability of long-term survival after surgery. However, the lack of external validation and an ordinary c-index restricted its clinical utility. Moreover, they demonstrated that the primary site of PDAC could significantly contribute to prognosis, suggesting that different primary sites of PDAC will show different prognosis. Therefore, it was reasonable and essential to evaluate the cancer prognosis separately for pancreatic head, body, and tail. The nomogram constructed in this study focused on the survival prognosis in PHDAC and was validated using both internal and external cohorts with perfect predictive performance. Most of the selected variables in the constructed nomogram were related to surgical treatment, indicating that the usage of this model could contribute to individualized therapy by assisting surgeons to distinguish patients with different prognosis after surgery. Additionally, the nomogram can be applied to identify high-risk PHDAC patients who may not benefit from upfront surgical treatment. Therefore, other non-surgical upfront treatment should be recommended, such as neo-adjuvant chemotherapy or immunotargeted therapy.

This study had several limitations. First, data were obtained from two medical centers, and the diversity of data from multiple centers was scarce. However, the volume of patients at our department is high, with 5,000–6,000 patients discharged each year, which can provide more data to justify our findings. Second, the time horizon of our data was 2012–2018 in the training cohort and 2002–2020 in the validation cohort. So, the sectional TJC type II/III/IV PHDAC patients before 2018 did not undergo preoperative chemotherapy when the 8th AJCC staging system was applied in clinical practice since 2018. Third, the TJC has been designed to optimize the surgical approach decision and has benefited surgical and prognostic outcomes to a certain extent, which can be observed in our daily surgical practice and in this analysis. However, the idiographic mechanism or decisive factor related to prognosis remains under investigation as another major research direction at our Biliary-Pancreatic disease research center. We will explore and address these issues in our future prospective real-world studies, and we believe that these studies would provide more encouraging results.

In conclusion, to the best of our knowledge, this study makes the first attempt to construct a nomogram for predicting survival after surgery in PHDAC with an adequate number of cases in the training and external validation cohorts. The predictive nomogram proposed in our study used clinical factors that can be easily determined and can objectively predict survival in PHDAC patients. It would aid surgeons in making prognosis-based decisions and selecting appropriate treatments for PHDAC patients who would benefit from PD. Additional studies would be required to determine whether the nomogram can also be applied to patients with other kinds of PDAC.

## Data Availability Statement

The raw data supporting the conclusions of this article will be made available by the authors, without undue reservation.

## Ethics Statement

The studies involving human participants were reviewed and approved by Institutional Review Board of Tongji Medical College, Huazhong Scientific and Technological University, China, and the Ethics Committee of the National Taiwan University Hospital. The patients/participants provided their written informed consent to participate in this study.

## Author Contributions

FP, TQ, and RQ designed the experiment. FP and TQ analyzed data. FP, TQ, MW, HW, and CD prepared the figures. FP and TQ wrote the manuscript. FP, TQ, C-HW, Y-WT, and RQ contributed to manuscript editing. All authors contributed to the article and approved the submitted version.

## Funding

This study was supported by grants from the National Natural Science Foundation of China (81402443 to FP; 81772950 to RQ; 81773160 to MW; 81702792 to SX; 81502633 to XL; 81602475 to XG; and 81874205 to FZ), the Hubei Natural Science Foundation (2017CFB467 to MW), the Tongji Hospital Science Fund for Distinguished Young Scholars (2016YQ08 to MW), and the Wuhan Applied Basic Research Project (2016060101010070 to RQ).

## Conflict of Interest

The authors declare that the research was conducted in the absence of any commercial or financial relationships that could be construed as a potential conflict of interest.

## Publisher’s Note

All claims expressed in this article are solely those of the authors and do not necessarily represent those of their affiliated organizations, or those of the publisher, the editors and the reviewers. Any product that may be evaluated in this article, or claim that may be made by its manufacturer, is not guaranteed or endorsed by the publisher.

## Supplementary Material

Supplementary Figure 1 Kaplan–Meier Curve Analysis. Survival curves stratified by different TJC types. [**(A)** Tongji cohort; **(B)** Taiwan cohort]. Patients with TJC Types I and II were better than those with TJC Type III/IV.

Supplementary Figure 2 X-tile analysis of the risk score in the training cohort.

Supplementary Figure 3 Kaplan–Meier curve analysis. Survival curves stratified by risk groups in patients at AJCC stages I, II, and III in the training cohort **(A–C)** and the validation cohort **(D–F)**.
