# Supplementary figures and images for: Development and Validation of a Nomogram to Predict Survival in Pancreatic Head Ductal Adenocarcinoma After Pancreaticoduodenectomy
Source: Front Oncol. 2021 Sep 29;11:734673. doi: 10.3389/fonc.2021.734673 (PMC8514110; doi:10.3389/fonc.2021.734673)

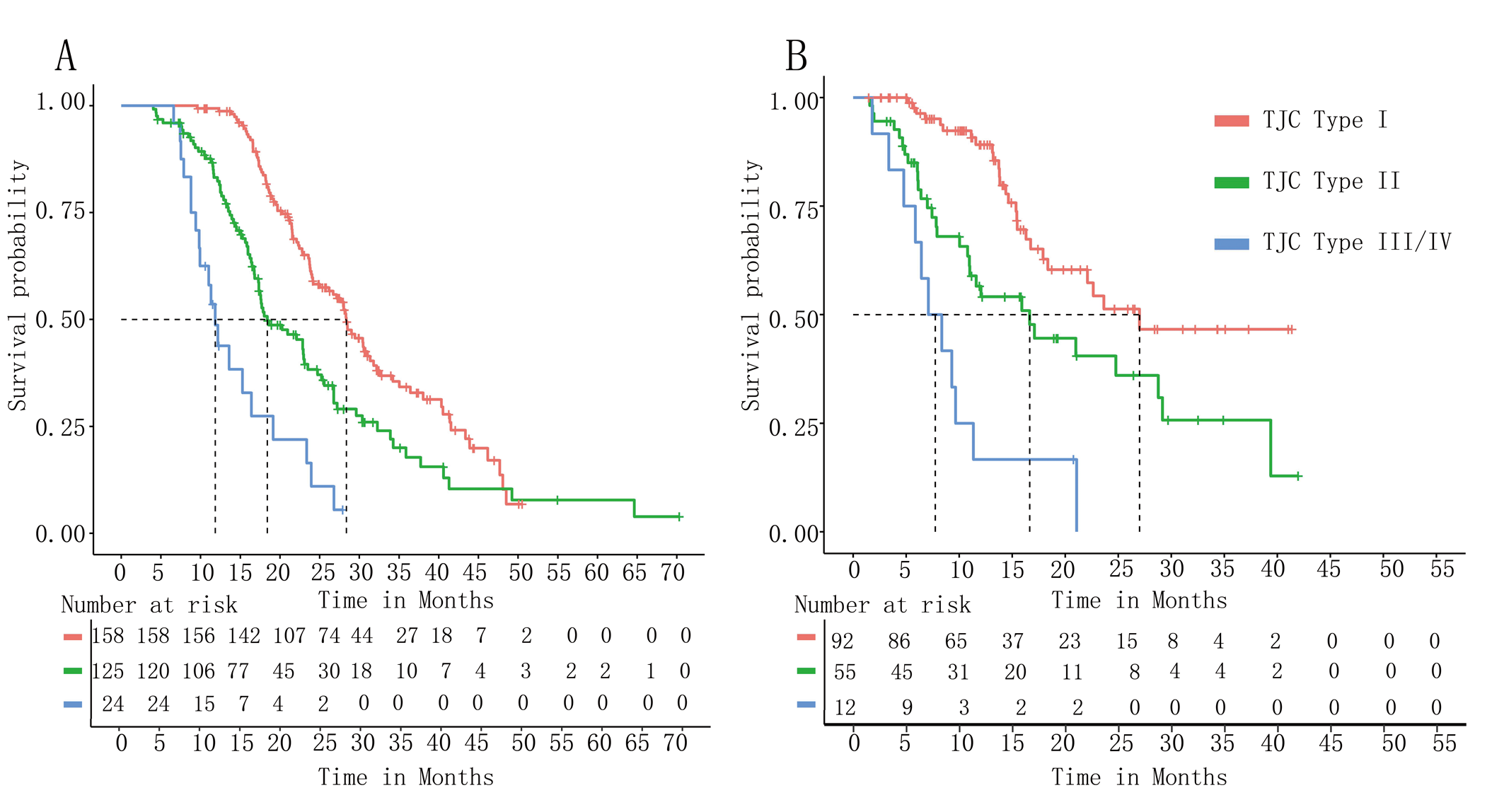

Supplement: Supplementary Figure 1 — Kaplan–Meier Curve Analysis. Survival curves stratified by different TJC types. [(A) Tongji cohort; (B) Taiwan cohort]. Patients with TJC Types I and II were better than those with TJC Type III/IV. [file Image_1.tif]

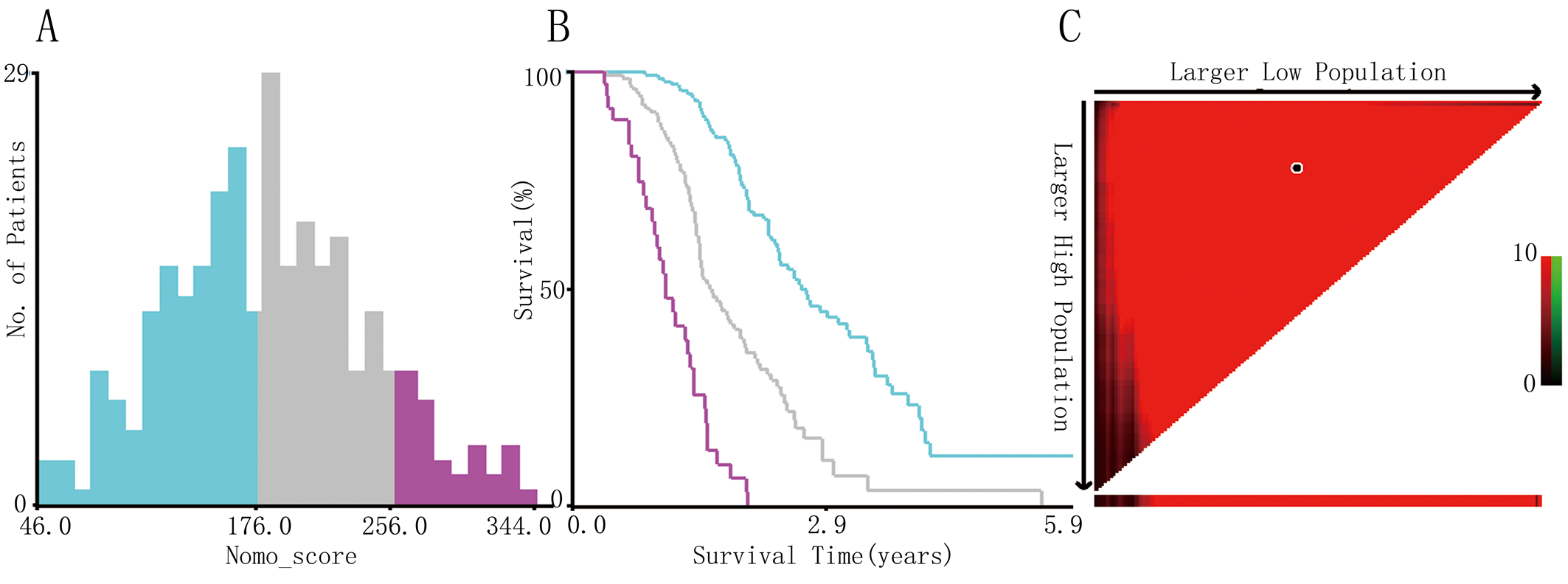

Supplement: Supplementary Figure 2 — X-tile analysis of the risk score in the training cohort. [file Image_2.tif]

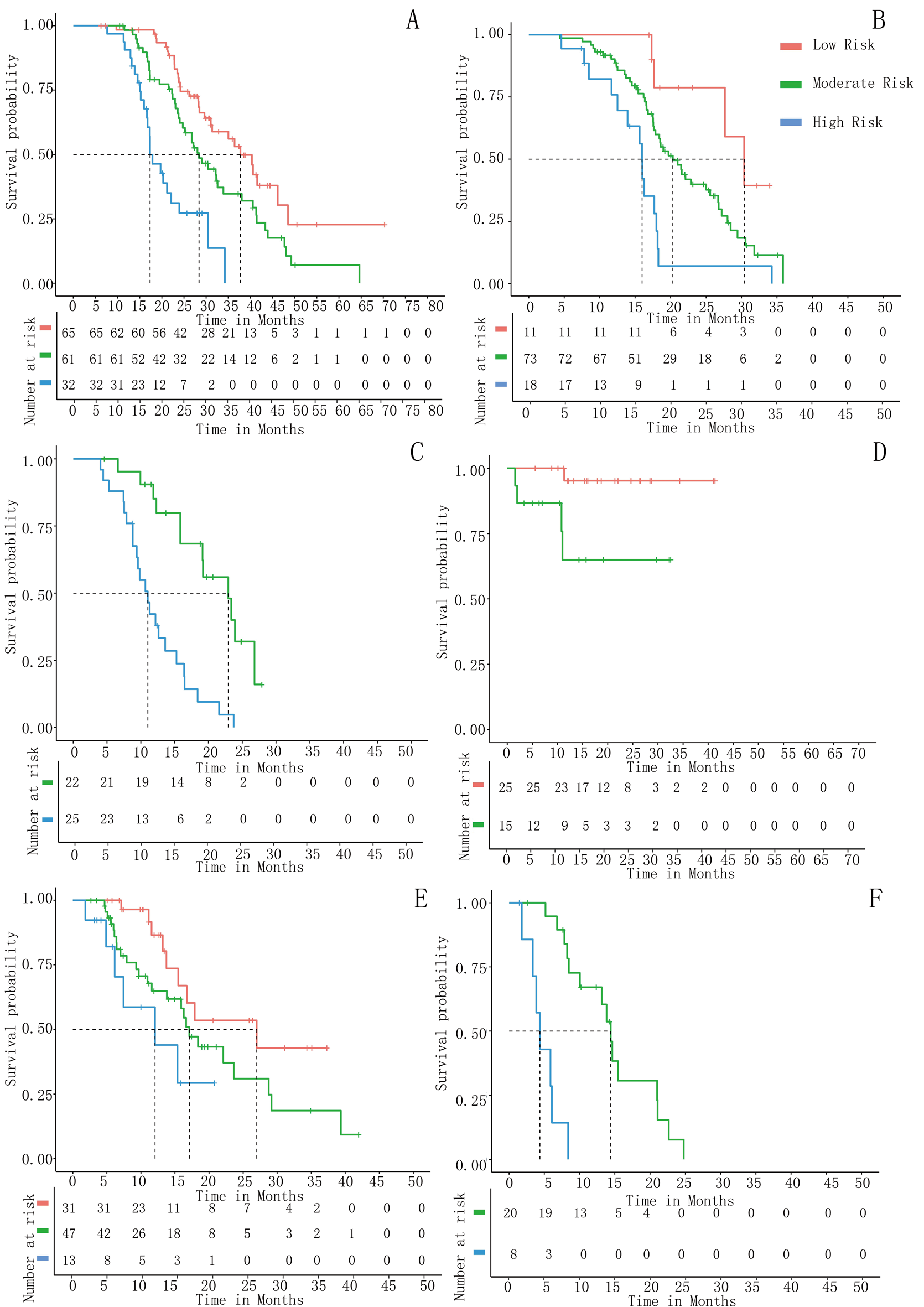

Supplement: Supplementary Figure 3 — Kaplan–Meier curve analysis. Survival curves stratified by risk groups in patients at AJCC stages I, II, and III in the training cohort (A–C) and the validation cohort (D–F). [file Image_3.tif]
