# Supplementary material for: Development and Validation of a Nomogram to Predict Survival in Pancreatic Head Ductal Adenocarcinoma After Pancreaticoduodenectomy
Source: Front Oncol. 2021 Sep 29;11:734673. doi: 10.3389/fonc.2021.734673 (PMC8514110; doi:10.3389/fonc.2021.734673)
Supplement: Supplementary file 4 [file Table_1.docx]

**STable 1. Supplemental Table 1. Point assignment and prognostic score**

| **Variable and prognostic score** | **Score** | **Estimated 12-month overall**  **survival (%)** | **Estimated 18-month overall**  **survival (%)** | **Estimated 24-month overall**  **survival (%)** |  |
| --- | --- | --- | --- | --- | --- |
| Age, years |  |  |  |  |  |
| 20–25 | 0 |  |  |  | |
| 26–30 | 5 |  |  |  | |
| 31–35 | 10 |  |  |  | |
| 36–40 | 15 |  |  |  | |
| 41–45 | 20 |  |  |  | |
| 46–50 | 25 |  |  |  |  |
| 51–55 | 30 |  |  |  |  |
| 56–60 | 35 |  |  |  |  |
| 61–65 | 40 |  |  |  |  |
| 66–70 | 45 |  |  |  |  |
| 71–75 | 50 |  |  |  |  |
| 76–80 | 55 |  |  |  |  |
| 81–85 | 60 |  |  |  |  |
| Preoperative CA 19-9, U/ml |  |  |  |  |  |
| <160 | 0 |  |  |  |  |
| 160–400 | 30 |  |  |  |  |
| ≥400 | 33 |  |  |  |  |
| Chemotherapy points |  |  |  |  |  |
| No | 0 |  |  |  |  |
| Yes | 72 |  |  |  |  |
| Tongji classification points |  |  |  |  |  |
| Type I | 0 |  |  |  |  |
| Type II | 10 |  |  |  |  |
| Type III | 99 |  |  |  |  |
| Type IV | 82 |  |  |  |  |
| Tumor differentiation |  |  |  |  |  |
| Well | 0 |  |  |  |  |
| Moderate | 48 |  |  |  | |
| Poor | 66 |  |  |  |  |
| T stage points |  |  |  |  |  |
| T1 | 0 |  |  |  |  |
| T2 | 18 |  |  |  |  |
| T3 | 61 |  |  |  |  |
| N stage points |  |  |  |  | |
| N0 | 0 |  |  |  | |
| N1 | 20 |  |  |  |  |
| N2 | 100 |  |  |  |  |
| Total prognostic score |  |  |  |  |  |
| <207 |  | >75 | >65 | >35 |  |
| 207–278 |  | 75–45 | 65–26 | 8–35 |  |
| >278 |  | <45 | <26 | <8 |  |

Abbreviation: CA 19-9, cancer antigen 19-9
