# Supplementary material for: Development and Validation of a Nomogram to Predict Survival in Pancreatic Head Ductal Adenocarcinoma After Pancreaticoduodenectomy
Source: Front Oncol. 2021 Sep 29;11:734673. doi: 10.3389/fonc.2021.734673 (PMC8514110; doi:10.3389/fonc.2021.734673)
Supplement: Supplementary file 5 [file Table_2.docx]

**STable 2. Supplemental Table 2. The Harrell’s C-index value in the training and validation cohorts**

|  | **Training** | **P value** | **Validation** | **P value** |
| --- | --- | --- | --- | --- |
| Nomogram | 0.79 (0.76–0.82) | Reference | 0.83 (0.78–0.87) | Reference |
| AJCC | 0.66 (0.62–0.70) | <0.001 | 0.67 (0.60–0.74) | <0.001 |
| Tongji classification | 0.58 (0.53–0.62) | <0.001 | 0.68 (0.62–0.74) | <0.001 |
| JPS | 0.58 (0.54–0.61) | <0.001 | 0.61 (0.55–0.66) | <0.001 |
| Age | 0.51 (0.47–0.56) | <0.001 | 0.55 (0.47–0.63) | <0.001 |
| Preoperative CA 19-9 | 0.54 (0.50–0.58) | <0.001 | 0.58 (0.51–0.65) | <0.001 |
| Diabetes | 0.53 (0.50–0.55) | <0.001 | 0.53 (0.46–0.59) | <0.001 |
| Tumor differentiation | 0.58 (0.54–0.62) | <0.001 | 0.60 (0.55–0.66) | <0.001 |
| T Stage | 0.55 (0.52–0.58) | <0.001 | 0.63 (0.57–0.70) | <0.001 |
| N Stage | 0.58 (0.54–0.62) | <0.001 | 0.66 (0.59–0.73) | <0.001 |

AJCC, American Joint Committee on Cancer; CA 19-9, cancer antigen 19-9; JPS, Japanese Pancreas Society.
